# An analysis of the medical specialty training system in Spain

**DOI:** 10.1186/s12960-015-0038-y

**Published:** 2015-06-02

**Authors:** José-Manuel Freire, Alberto Infante, Adriana Cavalcanti de Aguiar, Pilar Carbajo

**Affiliations:** National School of Public Health/Institute of Health Carlos III, Madrid, Spain; Institute for Communication and Information in Health, Oswaldo Cruz Foundation; Social Medicine Institute, Rio de Janeiro State University, Rio de Janeiro, Brazil; Ministry for Health, Social Affairs and Equality, Madrid, Spain

**Keywords:** Postgraduate teaching, Medical specialty training, Medical residency, Medical education

## Abstract

In this paper, we analyse the medical specialty training system in Spain (the so-called “residency system”). In order to do so, we a) summarize its historical evolution; b) describe the five major architectural pillars on which the system is currently based; c) analyse the special contract of the specialist-in-training; d) discuss the three major challenges for the medical specialist training future: the evolution and expansion of the residency system to other health professions, the issue of grouping specialties with a common core trunk and the continuity of the learning process; and e) draw four conclusions that may be relevant for those who are in the process of developing or revising their own medical specialization systems.

## Background

In Spain, as in every other country of the European Union, all university degrees and professional qualifications are publicly regulated. However, the particular importance of specialties in medicine, given their direct impact in professional practice and in the organization of patient care, made it necessary for a specific regulation that universities, formally “autonomous” since the 1990s, are responsible for the pre-graduate education leading to the medical degree in Medicine, with little input from the healthcare authorities.

Following graduation, most physicians seek specialization in a formally organized system that is regulated and run by the central government. The first initiative of formal regulation of the medical specialty training in Spain dates from 1955. Subsequent regulations recognized 33 medical specialties. Prior to this, the training of medical specialists was partially delegated to the university system and could be developed in rather lax conditions within the so-called “Institutes of Specialization” set up by certain chairs within the schools of medicine.

During the 1960s, the Social Security Health Services became the backbone of the Spanish healthcare system. Its rapidly expanding hospital network intended to cover the whole country with a system of modern hospitals. A system in such a rapid growth had an urgent need of doctors of all medical specialties, and therefore, the Social Security started to introduce in some of its hospitals a residency system (RS) thereby training its own doctors. At the end of the decade, doctors from several hospitals – H. de Basurto (Bilbao), H. Valdecilla (Santander), H. de la Santa Cruz y San Pablo (Barcelona), Fundación Jiménez Diaz and Clínica Puerta de Hierro (Madrid), among others – created the “Seminar of Hospitals with Post-graduate Medical Education” [1]. Its theoretical and conceptual recommendations established the basis of the current system of medical specialty training. By that time, the RS had already shown its effectiveness in the United States and elsewhere.

At the beginning of the 1980s the RS was conceived as system of learning through supervised and programmed professional practice for the specialist-in-training to acquire progressively the knowledge, skills, techniques and responsibilities needed to become an independent specialist. The basic principles of this system of professional specialist training were laid down in several official regulations issued by the Social Security Health Service authorities [2]. These early steps paved the way for a Royal Decree [3] that defined a new regulatory framework for medical specialties in Spain. Its main points were a) endorsement of the hospital residency system as the official route to medical specialization; b) definition of a new official medical specialties list (51 specialties); c) setting up of a National Council of Medical Specialties (NCMS) and a National Specialty Commission (NSC) for each specialty; d) establishing a fair, competitive and merit-based system of access to the RS, which included a nationwide annual competitive examination for applicants to the RS; and e) assignment of the main responsibility of training of medical specialists to the Ministry of Health (although the Ministry of Education retained the awarding of the certificates).

This regulation was a major step forward towards endorsing and consolidating the RS, but did not totally abolish the old system: a residual training for three medical specialties will remain until 2015 at the university specialty schools. However, as the Social Security was by far the main employer of doctors, the completion of a “residency” programme became a condition to work as a medical specialist in a public hospital.

On the eve of Spain’s entrance into the European Union (January 1986), a new Royal Decree [4] regulating medical specialties was approved. Its main points stated the following:

1. Forty-nine recognized medical specialties
2. A unified system of RS training which included the accreditation of postgraduate teaching hospitals (university specialty schools were retained for those specialties without hospital-based training).
3. Endorsement of the reforms introduced in 1978 (the National Council of Medical Specialties, National Specialty Commissions for each specialty, the nationwide competitive entry exam and the central role of the Ministry of Health).
4. The specific postgraduate training of general practitioners through the RS; the specialty was called Family and Community Medicine. There was a specific exam for family medicine every year during the adaptation period.

## Case study

Since 1986, the Spanish medical specialists training system rests on the five following pillars:

1. The National Council of Medical Specialties and the National Specialty Commission of each specialty
2. The training programme of each specialty.
3. The Accreditation of Hospitals and Clinical Services for Specialty Training.
4. The selection process of specialty candidates based on a national competitive exam.
5. The evaluation of the resident.

Each of them is examined in detail below. The legal and professional status of the resident physicians are also examined. The NCMS and the NSC presidents of the 47 NSCs form the NCMS. Each NSC has 11 physicians: 9 of them are meant to be of recognized prestige and are to be nominated by a) the Medical Scientific Societies of each specialty, b) the Spanish Organization of the Colleges of Physicians, c) the Human Resources Commission of National Health System and d) the Ministry of Education. Two members of each NSC represent specialists-in-training, and they are elected by their peers.

Overall, the NCMS and the NSCs form a group of more than 500 highly qualified doctors actively involved in the RS. The NSCs meet an average of two to three times a year, plus the meetings of the NCMS and those of its permanent commissions and different working groups. All these commissions perform very relevant functions for RS, since both the NCMS and NSCs are also advisory bodies of the Ministries of Health and Education. As such, they have certain statutory competences and responsibilities. Their professional and technical character, and the dedication and effectiveness demonstrated for several years, have indeed granted them great prestige and credibility.

### The training programme

The development of a training programme for each specialty is one of the aspects in which the work of the NSC has proven to be most useful and effective. All medical specialties have an official training programme (OTP) that defines the knowledge and skills deemed necessary for becoming a fully qualified medical specialist. These OTPs are published in the Official Bulletin of the State (BOE) and are subjected to a continuous reviewing process. Most of the current “medical specialty scientific societies” are medical specialty associations created to foster and share the base knowledge and skills of each specialty through peer-reviewed journals, scientific meeting, etc. They do not have any regulatory power or an official status, but they enjoy official recognition as seen here.

OTPs were published between 2005 and 2011. The latest update is the one of Medical Oncology, developed in response to European Union (EU) Directive 2005/36/EC (April 2013). For a long time, the Spanish OTPs were the only ones officially endorsed and approved within the European Union. The OTPs define the content and the training period of each specialty. The duration of the programmes is generally 4 years for most specialties, including Family and Community Medicine, and 5 years for the surgical specialties, internal medicine, cardiology, intensive care medicine and medical oncology.

### The accreditation of hospitals and clinical services for specialized training

Any health facility interested in the training of medical specialists needs to pass a formal process of accreditation, in which the NCMS and the NSCs have an important role. Accreditation is the process by which the education and health authorities verify that a given healthcare facility (and one or more of its units) meets the standards required for the award of the official authorization for the training of medical specialists. These requirements are approved following the proposal of the NCMS and are subjected to a periodical review process.

The accreditation process involves an audit and an inspection, a system that follows a well-established procedure. Teams of experts, none of whom belongs to the institution being audited, carry out inspections. These and audit reports are presented to the audit committee of the NCMS; this one may initiate, if needed, a process leading to the withdrawing of the accreditation.

**Fig. 1:**
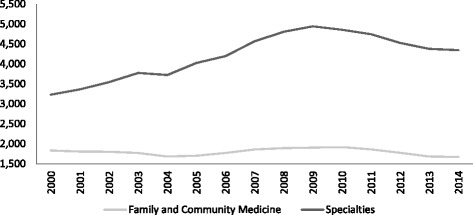
Evolution of admissions of specialist training in Spain. *Source*: Ministry of Health, Social Services and Equality. Family and Community Medicine is a specialty in Spain. The post-graduate training for general practitioners is carried out through this specialty

**Fig. 2:**
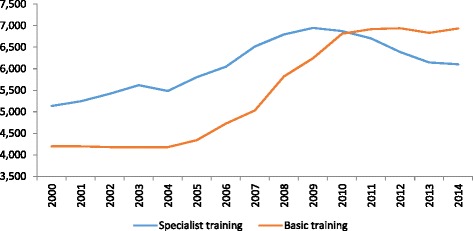
Evolution of medical schools intakes and specialist training posts in Spain

The accreditation granted to a specialty training unit includes the maximum number of training posts, in compliance with the EU requirements that require for a qualification of medical specialist to be accepted all over the EU, that training should be developed in posts “recognized by the competent Authorities”. In July 2013, 183 health centres were accredited, encompassing 2800 accredited services for specialists training. Fig. 1 shows the evolution during the last 10 years of the number of financed training posts of medical specialists in training (MIR), including Family and Community Medicine (general practice) and other medical specialties Fig. 2 shows the lack of correlation between the annual number of MIR training posts and the number of admissions in Spanish medical schools, a fact that could explain the high number of foreign medical graduates (mostly from Latin America) that have entered into the Spanish MIR system during this period.

### The selection process and the national competitive exam

The selection of candidates takes place every year, according to a calendar published well in advance. The first part of the process is the yearly approval of the number of financed training posts which is based on a) the existing accredited training capacity b) the national need of specialists considered by health authorities of the autonomous communities (regions) and c) 95 % vacancies are founded by the public sector and healthcare budgets of the autonomous communities (in 2012, the public cost of specialists training was 993.254 euros). In establishing the number of available specialty posts, the autonomous communities have thus an important role, together with the NCMS, the Human Resources Commission of the National Health Services Territorial Council and the Ministry of Health. Since 2014, the Ministry of Health can modify the number of posts proposed by the autonomous communities according to the overall needs of the country.

The annual approval of the national number of posts for medical specialty training is without doubt the main instrument of medical workforce planning in Spain; its importance cannot be overemphasized. The other relevant instrument is the annual number of openings available in the Spanish medical schools.

The selection procedure for access to specialty medical training is based on the constitutional principles of equality, merit and capacity for public employment. A national competitive exam takes place since in 1979, which was further regulated in 1989. The current regulation was established in 2003 [5], and the last update was in 2014 [5, 6]. The selection process is based on a test of knowledge, which weights 90 % in the final score, and in the candidates’ academic performance in the medical school, which weights 10 %. The whole evaluation procedure leads to a ranking of all candidates nationwide: those in the first positions have preference selecting the specialty and institution of their choice out of all national training posts available, leaving for those at the bottom of the ranking the remaining posts.

The exam is a multi-choice test of 225 items lasting a maximum of 5 h. Questions encompass all disciplines of the medical curriculum. More recently, the examination was revised to include both multiple choice questions and clinical reasoning problems and interpretation of laboratory results and clinical images. Since 2012, there is a minimum pass score to be eligible for choosing a training post, and 7 % of the posts are reserved for candidates with disabilities.

The selection process is designed to be transparent, fair and safe [7]. This implies open access to every step of the process and measures such as making public the criteria for the exam marking, publication of the correct answers, option to review the markings by those so wishing and the external design and elaboration of the exam questionnaire.

### Evaluation of the specialist in training

The last pillar of the specialty training system is the performance evaluation. The current general criteria of evaluation have been addressed in 2008 [8] as well as other aspects of the RS (teaching commissions of each accredited facility, tutoring, etc.) This regulation incorporated some suggestions made by the tutors [9].

The evaluation process involves both the head of the service (clinical unit) of the specialist-in-training and the tutor responsible for his/her supervision. The importance of a well-planned and personalized tutorial process must be emphasized [10]. Every year, the committee of evaluation of each specialty and training unit is required to evaluate the specialists-in-training in one of the following categories: failed, sufficient and outstanding. In case of a failed performance, a recuperation period of 3 months can be agreed upon, after which the resident is re-evaluated.

All policies and procedures are written in the “Resident’s Handbook”. This handbook is a document where the residents’ training milestones are recorded, including the following: participation in clinical activities and educational and research programmes and any other activity of interest. The recording of activities has to be countersigned by the staff specialist responsible for each activity with all applicable observations. The Resident’s Handbook is an element of great relevance, not only in the process of evaluation of the specialist-in-training but also as a portfolio for the recording of the training and professional curriculum.

Since 2008, the residents themselves evaluate the overall training process. This evaluation includes an annual survey of the residents’ satisfaction levels. Its purpose is to contribute to the development, improvement and quality control of the whole specialist training system. Therefore, the evaluation procedure is not “frozen”, it encompasses built-in feedback mechanisms for prompting the use of more specific evaluation procedures should it be required by any circumstance [11, 12]. These mechanisms might lead to the reviewing of training programmes themselves.

### The specialist-in-training: the resident physicians

Resident physicians in Spain have the status of professional employees – not at all students – and they bear an important proportion of medical work in hospitals and health centres, always under the supervision and guidance of fully qualified specialist physicians. They have a “special” contract, which includes full social security coverage for the period of their training. At the end of the residency, if they do not find a job, they are entitled to unemployment benefits as any other worker.

The work contract rights and duties of residents derive from the regulations of their specialty training [4, 5, 8], the regulation applicable to the work contract of trainees and the Guide for Specialized Training. The summary of the rights and duties of the residents-in-training includes the following:

1. The right and duty to follow the training programme, with progressive increase of the responsibility level, under the appropriate assistance and supervision.
2. To provide medical care to patients under the professional authority of the head of the unit and the training committee of the health facility.
3. The residence training is a full-time paid job with exclusive dedication to the healthcare institution where the training programme is developed; it is incompatible with any other professional activity.
4. The award of an official certificate of the completion of the training, following a positive evaluation.
5. Acceptance of the evaluation procedures established by the NCMS and NSC.

### Discussion and evaluation

The RS in Spain is very dynamic. In a broad perspective, it might be analysed, at least, from three different complementary approaches:

#### The evolution and expansion of the residency system to other health professions

Today, RS is employed for the training of medical specialists as well as for other health professionals, whose inclusion has been established by specific Royal Decrees: pharmacists (1982); radio-physicists (1997); clinical psychologists (1998); chemists, biologists and biochemists (2002); and nurses (2005). Consequently, the RS has been renamed “Specialized Health Training”, the National Commission of Medical Specialties became the National Commission for Health Sciences after the inclusion of new NSCs.

#### The issue of grouping specialties with a common core trunk

Article 19 of Law 44/2003 allows for the grouping of medical specialties, according to criteria of affinity. The creation of core-trunk specialties has the main purpose of reversing the current situation in Spain of a too-early specialization. This has been seen as narrowing the range of professional skills and competences, leading to the need of a greater number of specialists for a full coverage of clinical services, shaping accordingly the medical workforce planning, and leading to higher costs. The main aim, however, was not to set up new medical specialties based on broader competences, as is the case in the USA, but to define a period of common core training shared by all specialties of the same trunk during the early years of the training. This included also learning early on how to deal with health problems in a comprehensive manner and to work in a multi-professional team environment. Another objective was to facilitate the procedures for re-specialization within the same core trunk of those specialists wishing to be certified in a new specialty within the same core trunk, based on the evidence that this may appeal to many physicians during their career, thus providing the health system with greater flexibility according to future medical workforce needs.

This new approach to medical specialization was included in Articles 24, 25 and 29 of the aforementioned Law 44/2003. Those articles enable trainees to focus on medical areas related to the most up-to-date aspects of scientific developments within one or several specialties. The overall aim is that residents, at the completion of their specialty-training period, display a broader set of competences than is currently the case, with a better mix of general and specialized skills, becoming more able for life-long learning and continuous professional development. Medical specialties definition and boundaries is a turf of professional power clashes, so the process of defining core-trunk specialties has had a long and complex gestation period [13]. After much consultation and lobbying, a draft of Royal Decree regulating these issues was approved and was published in 2014: Royal Decree 639/2014, July 25 [14].

#### The continuity of the learning process

To meet with European Higher Education Space requirements (the so-called “Bologna Process”), a “final year of practice” ought to be included at the end of the undergraduate medical education [6, 15]. The inclusion of this final year started in the academic year 2008–2009 in all of the Spain’s medical schools. An important question, nevertheless, remains: how the contents of this “practical year” will fit in with the postgraduate medical specialty training programmes. Additionally, the Law 44/2003 (Article 33 and others) states that continuous medical education (CME) is a right as well as a duty for health professionals. This Law created a commission for CME comprised of representatives of the 17 autonomous communities and the Ministry of Health. This commission defines the framework for the CME, both with regard to the accreditation of the CME activities as well as to the accreditation of the institutions that provide such education. The autonomous communities are responsible for certifying the courses and the educational institutions within their territories. Spain is also working on the regulation of medical subspecialties, which will become specific areas of a specialty. Its training will be developed as a component of RS and similar to it.

Currently, medical professional re-certification is not mandatory, although some medical specialty scientific societies offer voluntary re-certification programmes for their members. Data on specialists re-certified or rejected or on the procedure and requirements are not available. More recently, the OMC (Spain’s National Organization of Medical Colleges) has launched a project to develop a voluntary re-certification mechanism for Spanish physicians [16].

## Conclusions

Most experts and observers acknowledge that the physician’s residency training scheme has been a major contributor to the development and good performance of the healthcare system in Spain [17, 18]. The overall high quality of the Spanish healthcare shown by many indicators, including national and international scientific medical publications [18], would not have been possible without the generations of physicians trained under the RS set up by the Social Security Health Service in the mid-1960s.

Long-term vision and continuous professional involvement have been essential in the design, implementation and continuous review and redesign of a system that works well, although this has not been an easy process. Spain has a highly decentralized health system, and the many actors involved represent an added source of complexity.

Nevertheless, the high level of commitment of relevant stakeholders (medical councils, specialty scientific societies, central and regional health and education authorities, etc.) increases the legitimacy of the whole process: all of them are periodically consulted and briefed about every step of the RS.

Ensuring the continuity of training since medical school to the end of a productive professional life, implementing the new “common core trunk” approach for RS and introducing a mandatory mechanism for health professional re-certification are probably the three biggest challenges for the Spanish National Health Services in this area.

In conclusion, the Spanish medical specialty training system is a good and resilient example of the governance of the process regulating medical professional “learning-by-doing”. It demonstrates how complex and important is the co-management of the whole process by all relevant stakeholders in sensitive issues of a profession such as medicine. In the authors’ view, it offers a number of lessons and experiences likely to be useful for other countries.

## Abbreviations

CME: Continuous medical education
BOE: Official Bulletin of the State
EU: European Union
MIR: Medical specialists in training
NCMS: National Council of Medical Specialties
NSC: National Specialty Commission
OTP: Official training programme
RS: Residency system
